# Could MicroRNAs Be Useful Tools to Improve the Diagnosis and Treatment of Rare Gynecological Cancers? A Brief Overview

**DOI:** 10.3390/ijms22083822

**Published:** 2021-04-07

**Authors:** Riccardo Di Fiore, Sherif Suleiman, Francesca Pentimalli, Sharon A. O’Toole, John J. O’Leary, Mark P. Ward, Neil T. Conlon, Maja Sabol, Petar Ozretić, Ayse Elif Erson-Bensan, Nicholas Reed, Antonio Giordano, C. Simon Herrington, Jean Calleja-Agius

**Affiliations:** 1 Department of Anatomy, Faculty of Medicine and Surgery, University of Malta, MSD 2080 Msida, Malta; sherif.s.suleiman@um.edu.mt; 2 Center for Biotechnology, Sbarro Institute for Cancer Research and Molecular Medicine, College of Science and Technology, Temple University, Philadelphia, PA 19122, USA; president@shro.org; 3 Cell Biology and Biotherapy Unit, Istituto Nazionale Tumori-IRCCS-Fondazione G. Pascale, I-80131 Napoli, Italy; f.pentimalli@istitutotumori.na.it; 4 Departments of Obstetrics and Gynaecology and Histopathology, Trinity St James’s Cancer Institute, Trinity College Dublin, 8 Dublin, Ireland; shotoole@tcd.ie; 5 Department of Histopathology, Trinity St James’s Cancer Institute, Trinity College Dublin, 8 Dublin, Ireland; olearyjj@tcd.ie (J.J.O.); wardm6@tcd.ie (M.P.W.); 6 National Institute for Cellular Biotechnology, Dublin City University, Glasnevin, 9 Dublin, Ireland; neil.conlon@dcu.ie; 7 Laboratory for Hereditary Cancer, Division of Molecular Medicine, Ruđer Bošković Institute, 10000 Zagreb, Croatia; maja.sabol@irb.hr (M.S.); pozretic@irb.hr (P.O.); 8 Department of Biological Sciences, Middle East Technical University, Ankara 06810, Turkey; erson@metu.edu.tr; 9 Beatson Oncology Centre, Gartnavel General Hospital, 1053 Great Western Road, Glasgow G12 0YN, UK; Nick.Reed@ggc.scot.nhs.uk; 10 Department of Medical Biotechnologies, University of Siena, 53100 Siena, Italy; 11 Cancer Research UK Edinburgh Centre, Western General Hospital, University of Edinburgh, Crewe Road South, Edinburgh EH4 2XR, UK; Simon.herrington@ed.ac.uk

**Keywords:** rare gynecological cancers, microRNAs, miRNAs, cancer stem cells, circulating biomarkers, extracellular vesicles, microRNA-based therapy

## Abstract

Gynecological cancers pose an important public health issue, with a high incidence among women of all ages. Gynecological cancers such as malignant germ-cell tumors, sex-cord-stromal tumors, uterine sarcomas and carcinosarcomas, gestational trophoblastic neoplasia, vulvar carcinoma and melanoma of the female genital tract, are defined as rare with an annual incidence of <6 per 100,000 women. Rare gynecological cancers (RGCs) are associated with poor prognosis, and given the low incidence of each entity, there is the risk of delayed diagnosis due to clinical inexperience and limited therapeutic options. There has been a growing interest in the field of microRNAs (miRNAs), a class of small non-coding RNAs of ∼22 nucleotides in length, because of their potential to regulate diverse biological processes. miRNAs usually induce mRNA degradation and translational repression by interacting with the 3′ untranslated region (3′-UTR) of target mRNAs, as well as other regions and gene promoters, as well as activating translation or regulating transcription under certain conditions. Recent research has revealed the enormous promise of miRNAs for improving the diagnosis, therapy and prognosis of all major gynecological cancers. However, to date, only a few studies have been performed on RGCs. In this review, we summarize the data currently available regarding RGCs.

## 1. Introduction

Gynecological cancers are cancers that arise in the female reproductive organs, encompassing ovarian, fallopian tubal, uterine/endometrial, cervical, vaginal and vulval cancers, and gestational trophoblastic disease [1]. Each gynecological cancer has its own signs, symptoms and risk factors. Gynecological cancers pose an important public health issue, with a high incidence among women of all ages [2]. Patients are often diagnosed at a late stage. This could be due to several reasons including lack of awareness of specific differential symptoms, improper screening and even misdiagnosis [3]. Late diagnosis, combined with limited treatment options for advanced gynecological cancers are major contributing factors to the high mortality, thus emphasizing the need for further advancement in the area. These issues are further exacerbated in the case of rare gynecological cancers (RGCs) [4].

Many gynecological cancers, for example malignant germ-cell tumors, sex cord-stromal tumors, gestational trophoblastic neoplasia, vaginal/vulvar carcinoma, and melanoma of the female genital tract, are uncommon and have different clinicopathological characteristics, thus implicating diverse molecular biological pathogeneses. These tumors are defined as “rare”, with an annual incidence of <6 per 100,000 women and cumulatively account for over 50% of gynecological cancers [5,6,7,8]. RGCs are generally associated with poor prognosis. Since these cancers are rare, patient management becomes difficult in terms of correct diagnosis and limited therapy options, and given the low incidence of each disease, this poses a major hurdle in the management of patients.

The field of miRNAs has been increasingly investigated because of their potential role in the regulation of different biological processes [9]. miRNAs are a class of non-coding RNAs that are approximately 20–22 nucleotides in length, and are involved in the regulation of gene expression. Usually, miRNAs induce mRNA degradation and/or translational repression by interacting with the 3′ untranslated region (3′-UTR) of target mRNAs. There are few cases of miRNAs interacting with different regions on genes including promoters. They have also been reported to be involved in the or activation of and regulation of gene transcription [10]. According to the latest miRbase [11], 38,589 hairpin precursors and 48,860 mature miRNAs have been reported for nearly 300 organisms. For the human genome, the current numbers are 1917 annotated hairpin precursors, and 2654 mature sequences [11]. Given the rate of discovery of new miRNAs, it is predicted that, in fact, miRNAs may regulate the expression of almost one-third of all human genes [12].

Recent research has revealed the enormous promise for miRNAs to improve the diagnosis, and management of all major gynecological cancers (cervical, endometrial and ovarian cancers) [1]. This is backed up by research on miRNAs in other cancers such as thyroid, breast and gastric cancer [13,14,15]. Numerous miRNAs are believed to influence multiple biological functions, leading to modulation of the tumor microenvironment, including stemness, growth, proliferation, invasion and metastasis [1]. In addition, miRNA signatures have been proposed as potential biomarkers that can be used for early detection of gynecological cancers, as well as predictors of response to ongoing therapies [1]. Based on the available and emerging data, miRNAs could impact future therapeutic strategies for ovarian, cervical, and endometrial carcinomas.

Almost 15 years have passed since the first publication on the aberrant expression of miRNAs in human epithelial ovarian cancer [16]. However, to date, only a few studies have been performed on RGCs. In this review, we summarize the data currently available, in order to assess the progress made to date.

## 2. Biogenesis and Function of miRNAs

**Figure 1:**
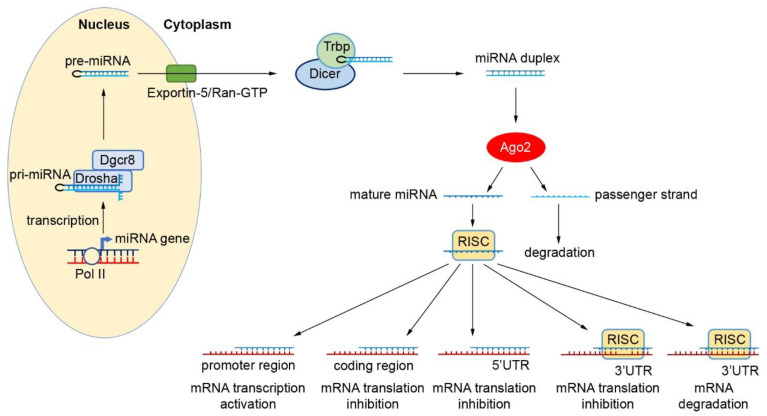
miRNA biogenesis and role in gene regulation. In the nucleus, primary miRNA (Pri-miRNA) is transcribed by RNA polymerase II (pol II). Pri-miRNA is cleaved by Drosha complexed with the protein ‘DiGeorge syndrome critical region 8′ (DGCR 8). Precursor miRNA (pre-miRNA) is released and transported to the cytoplasm by Exportin-5/Ran-GTP, a nuclear export factor. Here, a mature 17–25 bp miRNA duplex is generated through a series of cuts by RNase III endonuclease, Dicer/TRBP and Ago2. This miRNA duplex associates with the RNA silencing complex (RISC) to suppress target mRNAs by binding to the 3′ untranslated region (3′UTR) region. Binding to 5′UTR, 3′UTR and coding region could either inhibit the translation or degrade the mRNAs. Binding to the promoter region could activate the transcription.

In the search for novel diagnostic and therapeutic targets for cancer, miRNAs have become of significant interest, particularly because of their abundance and potential ease of detection in both tissue and plasma, and therefore they represent potential non-invasive molecular markers for cancer diagnosis and therapeutic response. miRNA biogenesis is a multi-step process that starts in the nucleus (Figure 1) [17,18,19,20]. Here, miRNAs are generally transcribed by RNA Polymerase II into long primary transcripts (pri-miRNA) that are further processed in the nucleus by Drosha/DGCR8 to form an intermediate structure called the pre-miRNA (precursor), made up of 60-70 nucleotides [17,21,22]. The nuclear export factor Exportin-5/Ran-GTP carries the pre-miRNA to the cytoplasm where, following a series of excisions by RNase III endonuclease, Dicer/TRBP and Ago2, a mature 17–25 bp miRNA duplex is generated. A helicase unwinds this miRNA duplex to form a mature single-stranded miRNA, which enters into the RNA-induced silencing complex (RISC). At this point, the complex is directed to target mRNA. It is thought that mature miRNAs regulate gene expression through the binding to the 3′ UTR of target mRNA, thus degrading mRNA or inhibiting translation. In the presence of distinct cofactors and conditions, miRNAs may be capable of activating gene expression directly or indirectly in response to different cell types [10,23,24]. Thus, this process allows the cell to respond rapidly to different cellular conditions due to the reversibility in their post-translational gene regulation. In addition, cells can produce miRNAs via several non-canonical processes. Some miRNA-like species, such as a subclass termed agotrons, are capable of bypassing particular steps of the canonical miRNA biogenesis pathway, escaping both Drosha and Dicer processing [25].

## 3. miRNAs as Novel Therapeutic Strategies

**Figure 2:**
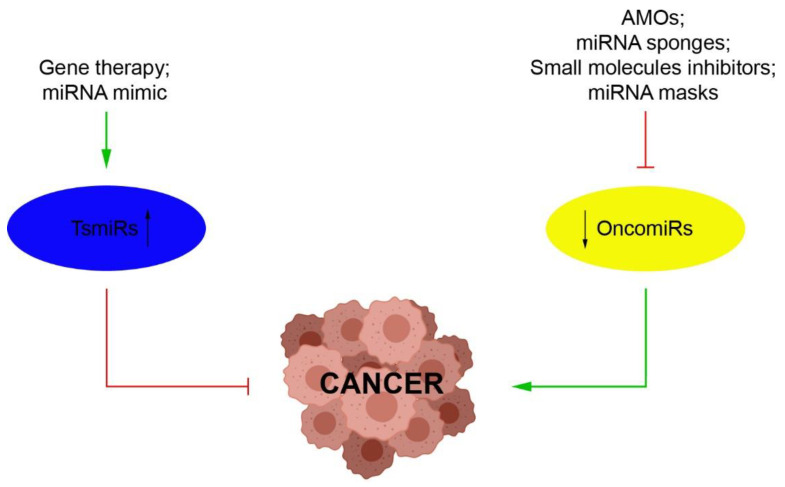
miRNA-based therapeutic intervention in cancer. Through inhibition of oncogenic miRNAs (Oncomirs), miRNA expression is modulated using small molecule inhibitors, anti-miR oligonucleotides (AMOs), miRNA masks/target protectors or miRNA sponges, or by reconstituting tumor suppressor miRNAs (TsmiRs), through delivery of miRNA mimics or gene therapy. BioRender has been used to create parts of this figure. (https://biorender.com, accessed on 26 March 2021).

The link between cancer and miRNAs was first reported almost two decades ago [26,27]. Studies in numerous human cancers have since confirmed that miRNAs are often associated with sites of chromosomal amplification or instability [28]. The aberrant expression of miRNAs has been linked to the stage, progression and metastasis of a wide variety of tumors [28,29]. In fact, miRNAs can function as tumor suppressors (TsmiRs) or tumor promoters (OncomiRs) [30,31]. In addition, miRNAs have also been shown to be involved in cancer stem cells (CSCs) and epithelial–mesenchymal transition (EMT), which are largely responsible for drug resistance and cancer metastasis [1,29]. Hence, targeting miRNAs holds the promise of being an effective option in the management of cancer, with possible therapeutic approaches including achieving “gain” or “loss” of miRNA functions in the tumor cells (Figure 2) [32]. For example, restoring the expression of tumor-suppressive miRNAs may be therapeutically beneficial [32]. In fact, synthetic miRNA mimics have already been used to restore the function of miRNAs in cancer cells [33]. In contrast, several approaches have been applied to achieve the downregulation of oncogenic miRNAs, including the use of miRNA sponges, small molecule inhibitors, anti-miRNA oligonucleotides (AMOs) and miRNA masking [34,35,36]. Overall, restoration or silencing of miRNA function is a promising strategy for cancer treatment. Furthermore, since miRNAs are also involved in radio- and chemo-sensitization of cancer cells, a combination of approaches may be developed for improved therapeutic outcome [37].

However, to date, there are several challenges that need to be overcome before miRNAs can actually be used as therapeutic agents. In fact, RNA oligonucleotides have certain features that complicate drug design and efficacy. Initial hurdles are RNA degradation by nucleases upon addition into biological systems and poor cell membrane penetration. These can potentially be dealt with by undertaking chemical modifications to the oligonucleotides, and using different delivery systems (e.g., liposomes, polymers, exosomes) to make up for their hydrophilic characteristics, negative charge and high molecular weight, which usually block nucleic acids from penetrating the cell membrane [38]. Other challenges include entrapment in the endosome, poor binding affinity for complementary sequences, poor delivery to desired target tissues; activation of innate immune responses and, last but not least, off-target and unwanted toxicities.

## 4. Dysregulation of miRNAs in Rare Gynecological Cancers

Emerging evidence demonstrates dysregulation of various miRNAs in gynecological cancers, suggesting pathobiological relevance [1]. Here, we highlight the ongoing challenges associated with the early diagnosis and effective treatment of specific RGCs and discuss how miRNAs may further improve the diagnostic, prognostic and therapeutic strategies for these tumor types (Table 1).

### 4.1. Tubo-Ovarian Cancer

Ovarian cancer is the leading cause of death in women who are diagnosed with gynecological cancer, and overall, it is the fifth most frequent cause of death in women. Ovarian cancer is divided into epithelial and non-epithelial subgroups. Epithelial ovarian cancer is classified according to histopathological appearance and clinical behavior and includes serous (low and high grade), clear cell, mucinous, endometrioid, carcinosarcoma and also fallopian tube and primary peritoneal cancer [39]. Non-epithelial ovarian tumors are relatively rare, but in total, they still account for approximately 10% of all ovarian malignancies. These are challenging from a diagnosis and management point of view, due to their rarity. Non-epithelial tumors include a large variety of different pathological types, including mesenchymal tumors (low- and high-grade endometrioid stromal tumors), mixed epithelial and stromal tumors (adenosarcomas and carcinosarcomas), pure stromal tumors (e.g., fibromas and thecomas), pure sex cord-stromal tumors (e.g., adult granulosa cell tumors and juvenile granulosa cell tumors), mixed-sex cord-stromal tumors (e.g., Sertoli–Leydig cell tumors) and germ cell tumors [40].

#### 4.1.1. miRNAs in the Rare Types of Epithelial Ovarian Cancers

Epithelial ovarian cancer is the most common group of ovarian cancer, however, it also includes the rare clear-cell, mucinous and low-grade serous carcinoma types [41]. High-grade serous carcinomas (which are not rare) almost always arise from the tube, and there is some evidence that low-grade serous carcinomas also do. Most of these types present at a low stage (by contrast with high-grade serous carcinoma) but they do on occasion present at a high stage. Especially when the diagnosis is at a late stage, although the initial treatment might possibly lead to a complete response, relapse is common with minimal response to chemotherapy [42].

Ovarian clear cell cancer (OCCC) diagnosed at an earlier stage tends to have a good prognosis as surgery is often curative. In advanced stages, however, it is more likely to be resistant to chemotherapy and has a worse prognosis. Due to the relative rarity of OCCC, there have been limited efforts in improving outcomes [4]. Similarities in molecular pathways exist among OCCC and clear-cell carcinoma of the kidney [43], where inhibition of angiogenesis, growth-factor signaling and mTOR pathways, might improve survival. Treatments that are used in the case of clear-cell carcinoma of the kidney, such as multikinase inhibitors (sunitinib, axitinib, sorafenib and pazopanib), temsirolimus, bevacizumab, and everolimus might have anti-tumor activity in OCCC, although preliminary clinical data from studies focusing only on OCCC are limited [44].

Loss of *ARID1A* and activation of *PIK3CA* are the most common somatic genetic alterations in OCCC (66.7% and 50% respectively), followed by mutations in *PPP2R1A* (18.8%) and *KRAS* (16.7%) [45]. Inhibition of the methyltransferase EZH2 and the administration of dasatinib and/or the HDAC6 inhibitor ACY1215 may represent novel treatment strategies for *ARID1A* mutated OCCC [46,47,48]. It has been shown that *ARID1A*-mutated OCCC cells have a specific sensitivity to small molecule inhibitors of the bromodomain and extra terminal domain (BET) family of proteins, to which BRD2 belongs, and which in turn causes a reduction in the expression of multiple SWI/SNF members including *ARID1B* [49].

EGFR inhibitors might be effective therapeutic agents, given that EGFR expression is detected in up to 60% of OCCCs [50]. In addition, high expression of mTOR has been reported in both early- and advanced-stage OCCC, with mTOR inhibitors being promising agents for treatment, especially in recurrent OCCC with cisplatin resistance [51].

Primary mucinous epithelial ovarian cancer (mEOC) accounts for less than 5% of epithelial ovarian cancers, with a decreasing incidence due to the fact that many cases that were previously diagnosed as primary mEOCs were actually metastases from other organs, mostly from the gastrointestinal tract. This highlights the importance of clinico-pathological review, because the pathology does not necessarily distinguish between primary and metastatic mucinous carcinomas, particularly if they have an upper GI phenotype. While it is recommended to treat mEOC with adjuvant carboplatin and paclitaxel, in-depth molecular characterization of mEOC suggests that trastuzumab (Herceptin) and HER2-targeted therapies might be an effective treatment as *HER2* is amplified or expressed in 19% [52] or 18.2% of these tumors [4], respectively.

Therefore, in order to improve prognosis through the development of a more specific treatment, thereby improving prognosis improved insight into the molecular characteristics of the different epithelial ovarian cancer subgroups. Several miRNAs, such as miR-509-3-5p, miR-509-3p, miR-509-5p, and miR-510, are differentially expressed in OCCC, high-grade serous ovarian carcinoma (HGSC) and ovarian surface epithelium (OSE), suggesting a carcinogenic role [53]. Furthermore, miR-424 has the capacity to suppress cell invasion and EMT in OCCC through downregulation of DCLK1, thus suggesting potential therapeutic targets [54]. Yanaihara et al. found higher levels of miR-9, miR-34a and miR-126 in OCCC, compared to HGSC [55]. Moreover, miR-9 overexpression may affect pathogenesis in OCCC by targeting E-cadherin and inducing EMT. In addition, it has been shown that miR-449 is under-expressed in OCCC [56] and that miR-29b signaling is involved in the sensitivity to chemotherapy in these cases [57]. Finally, Agostini et al. found that miR-192, miR-194, and miR-215 are upregulated in mEOC, suggesting that the miR192/215 family miRNAs may exert oncogenic functions in this tumor type [58].

#### 4.1.2. miRNAs in Nonepithelial Ovarian Cancers

Nonepithelial ovarian cancers including malignant germ-cell tumors and sex-cord stromal tumors are very rare and account for only 6% of all ovarian malignancies [59,60,61,62].

Malignant germ-cell tumors occur more commonly among women below 20 years of age and are often treated as their testicular counterparts. These tumors can be histologically classified as immature teratoma, dysgerminoma, yolk sac tumor, embryonal carcinoma, choriocarcinoma, mixed germ-cell tumor, malignant struma ovarii, teratoma with malignant transformation and gonadoblastoma [63]. Nowadays, with the use of platinum-based regimens, the five-year overall survival (OS) is estimated to be over 90% for early-stage tumors and above 75% for advanced disease [60]. The role of adjuvant chemotherapy is well established, with regimens such as BEP (bleomycin, etoposide, and cisplatin) being in routine use. However, in the case of relapsed ovarian germ-cell tumors, to date, there are no trials to suggest the benefits of a second-line therapy or the utility of high-dose chemotherapy to be followed by autologous stem cell transplant, as in testicular germ-cell tumors. Current practices include the use of TIP (paclitaxel, ifosfamide, and cisplatin), with extrapolation from treatment used for testicular germ-cell tumors, and even using more complex regimens containing combinations of cisplatin, methotrexate, bleomycin and vincristine, alternating with cyclophosphamide, actinomycin D, and etoposide [59]. Furthermore, targeted therapies that have been investigated consist of tyrosine kinase inhibitors (TKIs) (i.e., imatinib and sunitinib), trastuzumab (anti-HER2 monoclonal antibody) and antiangiogenic agents such as thalidomide and bevacizumab [64].

Granulosa cell tumors are a common type of malignant sex-cord stromal tumor and constitute about 5% of malignant ovarian tumors. There are two main distinct types: adult-type and juvenile granulosa cell tumors, which are different tumor types, but most data refer to adult-type tumors. Sertoli-Leydig cell tumors, steroid cell tumors, gynandroblastomas, and sex cord tumors with annular tubules are infrequently detected. Histologically, granulosa cell tumors are composed of granulosa cells, which secrete progesterone and estrogen [64]. Testing for the C134W *FOXL2* mutation is helpful in the diagnosis of adult-type tumors where the morphological appearances are not characteristic [65]. Granulosa cell tumors tend to have a slow progression and late recurrence.

In the case of women with advanced-stage or recurrent granulosa cell tumor, there is limited effectiveness of traditional chemotherapy [66]. The ongoing GOG264 trial (NCT01042522) is currently comparing the efficacy of carboplatin and paclitaxel versus (BEP) in advanced or recurrent sex cord-ovarian stromal cell tumors. Furthermore, targeted therapies such as vascular endothelial growth factor (VEGF) inhibitors, TKIs, and hormonal treatment have been investigated as therapeutic options for granulosa cell tumors [4].

Molecular pathogenesis of these tumor types is starting to be unraveled, especially in relation to the role of FOXL2 [67]. Chang et al. characterized miRNA expression profiles of some germ-cell tumors and sex cord-stromal tumors using small RNA sequencing [68]. Higher expression of miR-302c-3p, miR-372-3p and miR-373-3p, and lower expression of miR-199a-5p, miR-202-3p and miR-214-5p have been observed in malignant germ-cell tumors when compared to benign germ-cell tumors or sex-cord stromal tumors. In sex-cord stromal tumors, miR-513c-5p and miR-202c-3p were more abundant than in benign germ-cell tumors. Additionally, expression of Beclin 1 (*BECN1*), which is a target of miR-199a-5p, was shown to be higher in malignant germ-cell tumors than benign germ-cell tumors, which corresponds with their lower expression of miR-199a-5p.

Poynter et al. analyzed molecular signatures in dysgerminoma and yolk sac tumor, compared to adjacent tissue samples [69]. Differences in miRNA expression were observed, with miR-122, miR-302a, miR-302d, miR-371-5p and miR-373 showing elevated expression in one or more histologic subtypes. Correlations were also identified across six major hubs with higher expression in yolk sac tumor (miR-302b, miR-302a, miR-122 and miR 126; LEFTY1 and LEFTY2) compared with other germ-cell tumors. Cheng et al. validated six miRNAs (miR-29c-3p, miR-138-5p, miR-184, miR-204-5p, miR-328-3p and miR-501-3p) as novel markers for subtype classification in ovarian granulosa cell tumors with low levels of miR-138-5p correlating with early tumor stage, while low levels of miR-184 were linked with tumor recurrence in early-stage adult-type granulosa cell tumor patients [70].

A comparative study of miRNA regulation on FOXL2 between adult-type and juvenile-type granulosa cell tumors showed that reduction of the miR-17 family indirectly increased *FOXL2* mRNA expression [71]. Through miRNA profiling, juvenile- and adult-derived cell-lines have been shown to be biologically distinct, but this still needs to be addressed in vivo. Different studies by Tu et al. showed that miR-10a promotes granulosa cell tumor development via the PTEN-AKT/Wnt regulatory axis, while miR-126 is a tumor suppressor of granulosa cell tumor development via the regulation of *EGFL7* [72,73].

Mutations in the RNase IIIb domain of DICER1 are a common feature of nonepithelial ovarian tumors. These mutations lead to impaired miRNA biogenesis and thus disrupt miRNA levels. Mutations in both copies of *DICER1* result in the so- called DICER1 syndrome, and ovarian Sertoli-Leydig tumors are highly characteristic of this syndrome. Sertoli–Leydig tumors contain *DICER1* mutations in a high proportion of cases [74]. Hence, targeted therapies based on unique molecular pathways may be promising for better cure rates while reducing serious side effects.

### 4.2. Uterine Cancer

Uterine cancer can arise from both the endometrium and the myometrium. Uterine sarcomas, which arise from the middle muscular layer, are rare but are often aggressive and therefore need prompt diagnosis and treatment. Endometrial carcinoma is the most common type of gynecological cancer in women in developed countries, and it has been traditionally classified into two histological types. Type I tumors make-up 80–90% of endometrial cancers and are typically characterized by a low-grade endometrioid histology, on a background of atypical hyperplasia. These are characterized by estrogen and progesterone receptor positivity and, in most cases, have a favorable prognosis. Type II cancer occurs in 10–20% of endometrial cancers and is associated with typically high-grade non-endometrioid histology (serous endometrial cancer; clear cell endometrial cancer; uterine carcinosarcoma, UCS), arising in atrophic endometria. This is usually estrogen-independent and has a higher risk for metastases and less favorable prognosis [75].

#### 4.2.1. miRNAs in Uterine Sarcomas

Uterine sarcomas are aggressive mesenchymal tumors, with an incidence of 2–3% of all uterine malignancies [76]. There is a lack of consensus on risk factors and optimal treatment due to their rarity and diversity in their histopathology, thus generally leading to poor outcomes. Leiomyosarcoma (LMS), undifferentiated sarcoma and endometrial stromal sarcoma (ESS) are the predominant uterine sarcomas, with even rarer types such as rhabdomyosarcoma (including embryonal type in the cervix), and adenosarcomas [4,77].

ESS represents the second most common category of mesenchymal uterine tumors, in spite of accounting for less than 1% of all uterine tumors [76]. Endometrial sarcomas are further classified into low-grade ESS and high-grade ESS. Low- and high-grade ESS have been found to differ on a molecular level. Low-grade ESS is also a much more hormone-responsive and indolent tumor, whereas high-grade ESS is a more aggressive tumor [76]. This category was re-introduced into the WHO classification in 2014, and it is recognized that there is more than one molecular subtype of this tumor based on translocations [78,79]. Low-grade ESS are treated mainly by hysterectomy and bilateral salpingo-oophorectomy, but may include adjuvant radiation, hormonal treatment or aromatase inhibitors [80].

Patients with high-grade ESS have a higher mortality due to earlier and more frequent recurrences (often <1 year). Advanced or recurrent tumors must be treated aggressively with a combination of chemotherapyand radiation [81]. The role of maintenance therapy with treatments such as cabozantinib in high-grade uterine sarcoma are being investigated [82].

LMS is the most common uterine sarcoma. It occurs in women over 40 years of age and has a 50% 5-year survival rate when confined to the uterus. It appears that adjuvant chemotherapy or radiotherapy does not incur any added benefit [83]. Signs and symptoms of LMS resemble those of leiomyoma, which is more common, and hence the preoperative distinction between the two tumors may be difficult. In postmenopausal women who are not using hormonal replacement therapy, malignancy can be suspected by the tumor growth, although it is rare for a leiomyosarcoma to present as a rapidly growing tumor [76].

Treatment of leiomyosarcomas involves total abdominal hysterectomy with debulking of the tumor in case of local metastasis. Doxorubicin, docetaxel/gemcitabine, and ifosfamide are all possible treatment options for advanced or recurrent disease. Some tumors may respond to hormonal treatment [76]. Targeted therapies such as trabectedin and pazopanib have been investigated as treatment in advanced stage or metastatic leiomyosarcoma, with some degree of efficacy in disease control [76,84]. When compared to usual type leiomyomas, mutations in the cell cycle genes are more common in leiomyosarcoma samples. Hence, cell cycle-related mechanisms could be attractive targets for treatment for these rare tumors [85].

Undifferentiated sarcoma has no identifiable molecular marker and is essentially a diagnosis of exclusion. This is an aggressive cancer, and treatment options are deduced from experience with other high-grade soft tissue sarcomas. Clinical trials for the targeted therapies in soft tissue sarcomas are enrolling patients with uterine sarcoma [4]. While this enables the involvement of more patients into the clinical trials, it may hinder the specific analysis of these subtypes [4].

There is a lack of knowledge about the roles and molecular mechanisms of miRNAs in the physiological and pathological processes and about any correlation with prognosis and their potential to predict treatment outcome in patients with uterine sarcomas. Gonzalez Dos Anjos et al. analyzed miRNA expression profiles linked with the cancer-specific survival (CSS) of patients with uterine sarcomas [86]. In particular, in leiomyosarcoma, an association of lower CSS was found with the downregulation of miR-10a-5p and miR-125a-5p, and the upregulation of miR-34c-5p and miR-196a-5p. In endometrial stromal sarcomas, the down-regulation of miR-23-3p, let-7b-5p and let-7f-5p and the upregulation of miR-372-3p and miR-373-3p were associated with lower CSS. Higher survival rates were linked only to miR-138-5p upregulation. Patients with tumor metastasis and relapse had higher expression of miR-210-3p, miR-301a-3p and miR-335-5p. Finally, expression of miR-138-5p, miR-146b-5p, and miR-218-5p was linked with higher disease-free survival in treated patients. This suggests that these miRNAs represent potential prediction biomarkers for treatment response and prognosis in patients with such tumors.

Evaluation of the expression of 88 miRNAs known to be involved in LMS and ESS showed downregulation of miR-1, miR-23b, let-7c and let-7f in ESS in relation to the benign tissue. However, there were no statistically significant changes in miRNA expression levels between LMS tumors and controls [87]. In a molecular study that was conducted to compare the miRNA profiles of LMS and ESS and to compare the miRNA signatures of primary LMS, primary ESS and metastatic uterine LMS, 94 miRNAs were significantly differentially expressed in LMS and ESS [88]. Out of these miRNAs, 18 were overexpressed in LMS and 76 were overexpressed in ESS. In primary and metastatic LMS, 49 miRNAs were differentially expressed, with 45 being overexpressed in primary LMS and 4 overexpressed in metastases. These differing miRNA profiles in primary and metastatic LMS might help to improve the understanding of the progression of this malignancy. In LMS cells, five miRNAs exhibited an overexpression (miR-129-5p, miR-141-3p, miR-148a-3p, miR-202-3p and miR-203a-3p), and eight were downregulated (miR-1-3p, miR-21-5p, miR-27b-3p, miR-125b-1-3p, miR-140-5p, miR-152-3p, miR-485-5p and miR-495-3p). Of these, only three miRNAs showed significant expression in LMS (miR-1-3p, miR-202-3p and miR-7-5p). In addition, let-7 was also shown to be a potential prognostic biomarker in LMS [89,90].

In 2014, Guled et al. analyzed miRNA profiling on a series of LMS and undifferentiated pleomorphic sarcoma (UPS) samples, and in total, 38 and 46 miRNAs classified UPS and LMS samples, respectively, were compared to control samples. There was differential expression of miR-22, miR-126, miR-199a-3p, miR-199b-5p and miR-320a. In particular, miR-320a and miR-199-5p were highly expressed in LMS and UPS, respectively [91].

Finally, Stope et al. demonstrated that miR-1 is suppressed in LMS, compared to adjacent healthy tissue [92]. Moreover, in vitro studies suggested that miR-1 may be a pivotal tumor suppressor and represent a promising biomarker of diagnosis in LMS therapy. Overall, changes in miRNA levels are potentially important in terms of genomic copy number changes at miRNA gene loci and mRNA targets of these dysregulated miRNAs, which can have further implications in disease mechanisms.

#### 4.2.2. miRNAs in Uterine Carcinosarcomas

Uterine carcinosarcoma (UCS) is another rare gynecological cancer which accounts for less than 5% of uterine cancers [93]. It is a metaplastic carcinoma that is highly lethal with a 5-year survival rate of 33–39% [94]. Adjuvant treatment in case of metastasis largely includes the use of paclitaxel and carboplatin. To date, there is no trial that has shown an OS benefit from adjuvant radiotherapy or chemotherapy, even though most of these trials included a variety of gynecological sarcomas [95,96,97].

UCS is a biphasic tumor consisting of both mesenchymal (sarcomatous) and epithelial (carcinomatous) components. The mesenchymal component can resemble homologous histologic components commonly found in the uterus, or harbor heterologous components that are not normally native to the uterus, such as chondrosarcomatous or rhabdomyosarcomatous differentiation, and is by definition high-grade. The epithelial component is also high-grade and usually shows serous or endometrioid differentiation [98]. UCS shares mutational features similar to serous uterine carcinoma more frequently than endometrioid histologies, with extensive copy number alterations, and the majority harbor somatic *TP53* mutations. However, *TP53* mutation was found to be less common in “endometrioid” tumors [99]. UCSs are believed to have a monoclonal origin where, according to the conversion theory, carcinomatous subclones can undergo metaplastic differentiation to transform into sarcomatous cells late in tumorigenesis [100]. This conversion theory is backed by the fact that there is the co-expression of epithelial membrane antigens and cytokeratins in sarcomatous and carcinomatous cells, as well as identical patterns of X chromosome inactivation, concordance of *TP53* and *KRAS* mutations, and similar losses of heterozygosity between sarcomatous and carcinomatous components. Other frequent mutations have been found in *PIK3CA, FBXW7*, *TP53*, *KRAS*, *PPP2R1A*, and *PTEN*, similar to serous and endometrioid uterine carcinomas [100].

It has still not been determined how carcinomatous cells specifically undergo metaplastic differentiation. According to the conversion theory, it is believed that EMT allows the sarcomatous component to be derived from the carcinomatous component. EMT is a known process that causes cancer progression, metastasis and therapeutic resistance. The mechanism of EMT is also reversible, where mesenchymal–epithelial transition (MET) can decrease the progression of the tumor. Studies demonstrate that the over-expression of miR-200 in UCS cells induces a robust MET, leading to a decreased growth and aggressiveness of cells both in vitro and in vivo [100,101]. This suggests that advanced miRNA therapeutics using ectopic miR-200 expression may be a promising treatment for patients with UCS. A strong negative association has also been shown between expression of the miRNA-200 family and the levels of their promoter methylation. Therefore, epigenetic regulation of these miRNAs indicates a possible a mechanism for EMT in UCS [100].

Brunetti et al. performed molecular investigations on the expression status and mutations of the genes *FHIT, HMGA1/2*, *MTA1* and *LIN28A*; the pseudogenes *HMGA1P6* and *HMGA1P7*; and the miRNAs known to influence expression of these same genes in ovarian carcinosarcomas and UCS [98]. Mutations in *KRAS, PIK3CA*, and *TP53* were identified in UCS with a frequency of 6%, 31%, and 75%, respectively. In addition, an inverse correlation between downregulation of miRNAs such as miR16, miR26a, miR30c, miR214, let-7a and let-7d, and overexpression of *HMGA1/2*, and *MTA1*, were observed [98].

### 4.3. miRNAs in Vulvar Tumors

Vulvar carcinoma is also considered rare, accounting for 5% of female genital tract cancers, with the highest incidence in women aged 65 to 75 years. Patients with advanced or recurrent disease have a poor outcomes and increased morbidity [64,102]. Over 85% of cases are squamous cell carcinoma, and risk factors include human papillomavirus (HPV) infection, lichen sclerosus, and, especially in young women, vulvar intraepithelial neoplasia. Other histologic types also occur, including basal cell carcinoma, Bartholin gland carcinoma, extramammary Paget disease, sweat gland adenocarcinoma, adenocarcinoma of intestinal type, germ cell tumors, melanoma (see below) and mesenchymal tumors [63,102].

Treatment often consists of surgical management, which is then followed by radiotherapy with or without chemotherapy. Management of these patients is largely based on experience from the treatment of advanced cervical cancers [4].

Targeted therapies with potential benefit in vulvar carcinoma include TKIs such as erlotinib and cetuximab as EGFR genomic amplification and overexpression have been associated with poor survival in these patients. Erlotinib and combination cetuximab with chemotherapy in patients with recurrent vulvar carcinoma showed substantial response [103,104].

In addition, HER2 expression was also detected in extramammary Paget disease of the vulva. Although Paget disease is believed to have a good prognosis, recurrence is frequent. Hence, treatments targeted to HER2 may also benefit recurrent Paget disease of the vulva with HER2 overexpression [105].

High-risk HPV infection is related to vulvar intraepithelial neoplasia, basaloid, and warty carcinomas. In a retrospective study, p16 immunohistochemistry was positive in 166 of 550 tumors (30.2%) and p53 staining in 187 of 597 tumors (31.3%) [106]. Dysregulated cell cycle markers including increased expression of cyclin D1, and cyclin A has also been shown in vulvar carcinomas. These markers are related to poor clinical outcomes. Therefore, targeted agents for these molecular pathways, including a therapeutic HPV vaccine, are potential treatments for vulvar carcinoma [64].

Currently, there is limited information regarding the expression of miRNAs in vulvar carcinoma. de Melo Maia et al. characterized microRNA profile in vulvar tumors, correlating it with clinical and histopathologic data, and the occurrence of HPV infection [107]. There were 25 differentially expressed miRNAs between HPV-negative and HPV-positive groups, and 79 differentially expressed in tumors when compared to normal samples. Moreover, downregulation of both miR-19-b1-5p and miR-223-5p correlated with the presence of lymph node metastasis. Furthermore, downregulation of miR-19-b1-5p and miR-100-3p were associated with vascular invasion. In addition, overexpression of miR-133a and miR-519b were linked with advanced FIGO stage.

Yang and Wu investigated the mechanism of action of miRNAs in vulvar squamous cell carcinoma (VSCC) [108]. Altered expression of 157 miRNAs was detected in this type of carcinoma, with upregulation of miR-182-5p, miR-183-5p and miR-590-5p, and downregulation of miR-103a-3p, miR-107 and miR-603. There was a positive relationship between lymph node metastasis and miR-590-5p expression. Finally, upregulation of miR-590-5p may promote cellular malignant behavior via the target gene *TGFβR II*. In another study, there was an increased level of expression of miRNA-4712-5p in VSCC, promoting proliferation and invasion, by affecting PTEN and its downstream p-GSK3β, p-AKT, and cyclin D1 signaling pathways [109]. It has also been shown that miR-3147 serves as an oncomiR in VSCC via suppression of Smad4 [110]. These findings suggest future clinical applications related to miRNA deregulation in vulvar carcinoma.

### 4.4. miRNAs in Melanoma of the Female Genital Tract

Malignant melanoma, which overall accounts for around 1% of all cancers, is a malignant neoplasm of the skin and mucous membranes. The mucosal malignant melanomas, which are rarer and account for 0.03% of all cancers, may occur in various sites including the conjunctiva, oral cavity, esophagus, anus, and even the gynecological tract [111]. In fact, in women, 3% to 7% of all cases of mucosal malignant melanoma develop within the genital tract, mainly in the vulva and vagina. However, primary malignant melanoma of the uterine cervix is even rarer, with a five times lower incidence than primary vaginal or vulva cases of malignant melanoma [112]. Radical hysterectomy with regional lymphadenectomy and/or concurrent chemoradiation therapies are generally recommended, but the prognosis is usually poor and unpredictable. This is because there has been no absolute agreement on comprehensive treatment to date, due to its rarity and difficulty in diagnosis.

Recently, DiVincenzo et al. investigated miRNA expression profiles in melanomas originating from gynecological sites, such as cervix, vulva and vagina [113]. When comparing miRNA expression in vaginal melanoma to normal adjacent vaginal mucosal tissue, 25 differentially expressed miRNAs, were found. Moreover, 45 differentially expressed miRNAs were identified between vulvar melanoma and primary cutaneous melanoma, among which three demonstrated a decrease in expression in vulvar melanoma (miR-200a-3p, miR-200b-3p and miR-494-3p), and 44 demonstrated an increase in expression (including miR-17-5p, miR-146a-5p, and miR19b-3p). Among these differentially expressed miRNAs, both miR-17-5p and miR-146a-5p have been experimentally validated as direct or indirect regulators of PD-L1 expression in melanoma [114,115]. Furthermore, pathway analysis for differentially expressed miRNAs in vulval and vaginal melanoma has shown significant enrichment of 30 and 35 pathways, respectively, each including TGF-β signaling. In these cases, 57 genes in the pathway are validated targets of 13 differentially expressed miRNAs in vaginal melanoma, and 59 genes in the pathway are validated targets of 17 differentially expressed miRNAs in vulvar melanoma. These results indicate that miRNAs have an important role as potential regulators of gene expression in vaginal and vulvar melanomas, thus contributing to tumor progression.

### 4.5. miRNAs in Gestational Trophoblastic Disease

Gestational trophoblastic disease (GTD), which has an incidence of 2.0 per 1000 pregnancies refers to abnormal trophoblastic proliferation leading to a broad spectrum of lesions ranging from the benign, to premalignant, hydatidiform mole, through to gestational trophoblastic neoplasia (GTN), which encompasses the aggressive invasive mole, choriocarcinoma, placental site trophoblastic tumor and epithelioid trophoblastic tumor [78,116,117]. GTN is also referred to as persistent trophoblastic neoplasia (PTN) because it may arise after a normal term or preterm pregnancy, a molar pregnancy, abortion, or even an ectopic pregnancy. Although patients with GTN generally show a good response with more than 90% cure rate following chemotherapy, around 4% of cases would succumb to the disease [118].

The exact molecular mechanisms of the etiopathogenesis of GTD are still unclear. Human chorionic gonadotropin (HCG) is associated with excessive trophoblastic proliferation and can act as an angiogenic factor during implantation of molar pregnancy [119]. Thus, high HCG concentrations may increase the risk of persistent GTN. Hence, the efficacy of antiangiogenic agents should be explored for GTN.

Although more is now known regarding placenta-associated miRNAs, there is a lack of information regarding their role in the pathogenesis and progression of GTD. In complete hydatidiform mole, there is dysregulation of miR-517a, miR-517b, miR-518b, and miR-519a [120]. In cells derived from choriocarcinoma, miR-371a-5p and miR-518a-3p regulated different pathways related to tumorigenesis and metastasis [121]. These results may offer new clues to the proliferation and metastasis of GTD and may even provide possible diagnostic biomarkers for GTN.

It has been shown that miR-181b-5p, miR-181d-5p and miR-371a-5p are the most significantly altered miRNAs which are associated with progression to GTN [122]. Finally, other studies showed that miR-21 is involved in proliferation, migration, and invasion of choriocarcinoma cells, while miR-34, miR-196b and miR-199b may be tumor suppressors in choriocarcinoma [123,124,125,126].

## 5. Circulating miRNAs as Potential Biomarkers

To date, miRNAs have been detected in body fluids such as plasma/serum, saliva, cerebrospinal fluid, breast milk, pleural effusion, ascites, urine and vaginal discharge. This presents an opportunity as a non-invasive liquid biopsy approach for the diagnosis of a wide range of cancers [127,128,129]. Thus, miRNAs present in these fluids may serve as biomarkers offering easy and rapid non-invasive tests [130,131,132,133]. Additionally, extracellular miRNAs can be delivered to target cells by binding to proteins, such as argonautes [134] or via vesicles, such as exosomes, acting as endocrine, autocrine, and/or paracrine regulators and modulators of cellular activities [135]. This suggests that miRNAs may have hormone-like activities. Therefore, extracellular and circulating miRNAs can serve as biomarkers for diseases, as well as a means of intercellular communication.

Zhang et al. demonstrated through high-throughput sequencing, that plasma exosomes from women with ovarian cancer and healthy controls differently expressed miRNAs [136]: 31 were found to be downregulated and 34 upregulated. miR-99b-5p, miR-122-5p and miR-185-5p were significantly decreased, and miR-93-5p, miR-106a-5p and let-7d-5p expression levels were significantly increased, in patients with ovarian cancer compared with healthy women. Another study where circulating miRNA profiling was carried out in plasma samples of ovarian cancer patients, a variety of differentially expressed miRNAs were identified as possible biomarkers for the diagnosis, e.g., miR-19b-3p, miR-26b-5p, miR-125a-3p, miR-144-3p, miR-337-5p and miR-500a-5p [137]. However, to date, there are few studies on potential miRNAs as biomarkers for the diagnosis and prognosis of patients with RGCs (Table 2).

In 2014, Chao et al. analyzed the sera of patients with clear cell carcinoma and found that, in a set of 11 pairs of pre- and postoperative sera, the levels of four miRNAs (miR-130a, miR-138, miR-187 and miR-202) were higher in the sera of preoperative patients [138]. In addition, miR-130a remained consistent during the different time points in seven of the 10 patients during clinical follow-up. This suggests that miR-130a may be a useful serum biomarker for detecting the recurrence of OCCC.

Murray et al. showed that there were elevated levels of all eight main members of the miR-371∼373 and miR-302 clusters in the serum of a four-year-old child at diagnosis of yolk sac tumor [139]. Moreover, miRNA levels returned to normal during the clinical follow-up, with kinetics similar to a conventional marker α-fetoprotein. This study indicates that miR-371∼373 and miR-302 clusters could be promising candidate biomarkers for disease monitoring in malignant germ-cell tumors. A serum panel of choriocarcinoma-specific “chromosome-19-microRNA-cluster” (C19MC) microRNAs have been identified and were highly elevated at diagnosis but dropped rapidly upon starting treatment and normalized prior to the start of the second full chemotherapy course [140]. At diagnosis, the same authors also reconfirmed serum elevation of the previously identified marker of malignant germ-cell tumors, miR-371a-3p. Thus, these circulating microRNA markers seem to reflect choriocarcinoma disease activity more accurately than serum hCG, thus having potential in assisting clinical decision-making.

Circulating miRNAs were also investigated as potential biomarkers for leiomyosarcoma (LMS) [141]. The optimal model consisted of two miRNAs (miR-191-5p and miR-1246), with an area under the receiver operating characteristic curve for identifying LMS of 0.97 (95% confidence interval, 0.91–1.00). Seven serum miRNAs, namely miR-191-5p, miR-451a, miR-1246, miR-4430, miR-4485-5p, miR-4635 and miR-6511b-5p, were identified as a promising diagnostic model for LMS.

Finally, there is a significant decrease in plasma levels of miR-520b, -520c-3p and -520f in patients with complete hydatiform mole after evacuation [142]. Interestingly, in GTN patients, these three miRNAs tended to have a similar variation to serum hCG concentration [143].

The combination of multiple circulating miRNAs may be promising biomarkers for the diagnosis of gynecological cancers, including RGCs. However, inconsistent results of different study designs hamper the applicability of these findings as robust biomarkers. Therefore, further studies are required to validate these results.

In addition, 3′-UTR length isoform diversity is another issue in miRNA based gene regulation in cancers. 3′-UTR *cis*-elements recognized by miRNAs and/or RNA-binding proteins have a significant impact on the fate of mRNAs [144]. Alternative polyadenylation and/or splicing alters the 3′-UTR lengths in normal tissues and in cancer cells [145,146,147,148,149]. These 3′UTR isoforms are generally tissue and cancer-type-specific and hence have been suggested as potential biomarkers with prognostic potential [150,151,152]. Functionally relevant 3′UTR shortening or lengthening events may alter the miRNA binding landscape in cancer transcriptomes. Isoforms with different 3′UTR lengths are likely to be targeted differently by miRNAs, adding an extra level of complexity with implications at the translation step. Currently, there are few studies on ovarian cancers; hence our understanding of the 3′UTR diversity in gynecological cancers is very limited [152,153]. As transcriptome-level complexities are beginning to be investigated in gynecological cancers, a more comprehensive view of miRNA-based regulation is likely in the near future.

## 6. A Brief Overview on miRNAs and Their Regulated Targets in RGCs

The little evidence to date for miRNA functional targets is largely derived from reporter assays in combination with the cellular effects of modulation of miRNA expression in cell culture. We will proceed with describing miRNAs that may represent novel potential therapeutic targets for RGCs.

### 6.1. Up-/Down-Regulated miRNAs and Their Roles in RGCs

Several miRNAs that are overexpressed in RGCs have been shown to have oncogenic roles in vitro, as well as defined molecular targets that they regulate (Table 3). miR-9 [55], miR-10a [73], miR-21 [126], miR-590-5p [108], miR-3147 [110] and miR-4712 [109] have been described as “oncomiRs” across multiple mammalian cell types, which is consistent with their role in RGCs. In addition, underexpressed miRNAs in RGCs such as miR-34 [124], miR-126 [72] and miR-196b [125] act as tumor suppressors.

#### 6.1.1. miR-9 Induces EMT by CDH1 Targeting

Depending on the tissue type, miR-9 can act as a tumor suppressor or as an oncomiR. Similar to previous studies describing many other types of cancer [154], Yanaihara et al. [55] observed that there is an increased miR-9 expression in OCCC. In addition, luciferase-based assays have demonstrated direct binding between miR-9 and E-cadherin, which is a tumor suppressor protein encoded by the *CDH1* gene. The loss of its expression in association with EMT occurs frequently during tumor metastasis [155]. Moreover, miR-9 knockdown also limits invasion and migration while upregulating E-cadherin expression. This suggests that aberrant miR-9 expression might play an important role in EMT activation in OCCC cells via direct binding to and downregulation of E-cadherin. Therefore, miR-9 upregulation may be involved in OCCC pathogenesis by inducing EMT through E-cadherin modulation. Accordingly, miR-9 may be a promising therapeutic target strategy for OCCC [55].

#### 6.1.2. miR-10a Promotes Tumorigenesis by Regulating PTEN, Akt and Wnt Pathways

In patients with acute myeloid leukemia, miR-10a acts as an oncomiR via its repression of the p53/Rb network [156]. The microRNA-10 family could disturb the normal development of granulosa cells during follicle formation, and there is a strong miR-10a signal in tissues from malignant granulosa cell tumor patients [157]. Moreover, in vitro, forced expression of miR-10a promotes cell proliferation, invasion, migration, ovarian hormone production, and repressed anticancer drug-induced apoptosis. The oncogenic role of miR-10a was also validated in vivo. Interestingly, *PTEN*, a well-known tumor suppressor, was identified as a direct functional target of miR-10a, and AKT/Wnt as an associated oncogenic pathway of miR-10a in cancerous granulosa cells. These results demonstrate that miR-10a can promote granulosa cell tumor development via regulating *PTEN*, Akt, and Wnt pathways [73].

#### 6.1.3. miR-21 Targeted PDCD4 and PTEN Genes

miR-21 is one of the most commonly upregulated miRNAs in different types of malignant tumors [158]. Moreover, miR-21 is implicated in various processes involved in carcinogenesis such as proliferation, invasion, and metastasis. Meanwhile, miR-21 also participates in the regulation of multiple signaling pathways such as Nanog/STAT3, PI3K/Akt and PDGF pathways [158]. The regulatory function of miR-21 depends on its target genes such as *PTEN* and *PDCD4* (programmed cell death 4), which are both tumor suppressors [158].

Wang et al. [126] demonstrated the miR-21 could promote proliferation, invasion and migration of choriocarcinoma cells. Furthermore, miR-21 can activate the Akt pathway, negatively regulates *PDCD4* and *PTEN* and targets *PDCD4* in choriocarcinoma cells. This suggests that miR-21 is responsible for the aggressive phenotype of gestational trophoblastic disease and may have a potential diagnostic and therapeutic role to play in this condition.

#### 6.1.4. miR-590-5p Promotes Cellular Malignant Behaviors via the Target Gene TGFβRII

It has been reported that miR-590-5p promotes proliferation and invasion in human hepatocellular carcinoma cells via the direct targeting of *TGF-βRII*, which in turn plays an important role in cell growth and cancer development [159]. Yang and Wu found a positive relationship between miR-590-5p expression and lymph node metastasis [108]. Furthermore, they showed that miR-590-5p plays an oncogenic role in VSCC by promoting cell proliferation and migration through the manipulation of *TGFβRII* expression. This suggests that miR-590-5p may be a critical therapeutic target in VSCC.

#### 6.1.5. miR-3147 Regulates SMAD4

It has been shown that there is upregulation of miR-3147 in cervical squamous cancer and melanoma [160,161]. In VSCC, the expression of miR-3147 is markedly upregulated and the increased expression of miR-3147 is positively associated with the depth of invasion (4). In addition, miR-3147 regulates *SMAD4* by directly binding to its 3′ untranslated region. These results indicate that miR-3147 may have an oncogenic role in VSCC by targeting *SMAD4*, and miR-3147 may represent a novel potential therapeutic target for VSCC [110].

#### 6.1.6. miR-4712-5p Regulates PTEN and Affects Its Downstream p-AKT, p-GSK3β and Cyclin D1 Signaling Pathways

Yang et al. [109] investigated the role of miR-4712-5p and its regulatory mechanism in VSCC, and found increased levels of miR-4712-5p both in VSCC tissues and the A431 cell line. Moreover, miR-4712-5p overexpression promotes proliferation and invasion of VSCC cells. Luciferase-based assays have also demonstrated direct binding between miR-4712-5p and *PTEN*. In addition, miR-4712-5p overexpression increased phospho-AKT (p-AKT) and cyclin D1 expression, whilst there was a decrease in PTEN and phospho-GSK3β (p-GSK3β). Therefore, miR-4712-5p can reduce the expression of PTEN, further affecting its downstream p-AKT, p-GSK3β and cyclin D1 signaling pathways, promoting the proliferation and invasion of VSCC.

#### 6.1.7. miR-126 Regulates EGFL7

Epidermal growth factor-like domain-containing protein 7 (EGFL7) has been shown to be a critical oncogene in various types of cancer [162,163,164,165]. Notably, EGFL7 is highly expressed in patients with EOC, and its expression has been correlated with a poor prognosis [166]. In addition, EGFL7 also serves as a potential predictive marker of chemotherapy for cervical cancer [167].

Methylation-associated silencing of miR-126 and its host gene *EGFL7* has been demonstrated in pleural mesothelioma [168], suggesting an association between *EGFL7* and miR-126. Due to the similarities between granulosa cell tumors and those of mesothelial lineage, Tu et al. [72] showed that miR-126 constrains the tumorigenesis of granulosa cell tumors via directly targeting *EGFL7* and suppressing the phosphatidylinositol 3-kinase/ATK (PI3K/AKT) pathway. This suggests that miR-126 may be utilized as a prognostic marker or a therapeutic target for granulosa cell tumor treatment.

#### 6.1.8. miR-34a and miR-196b Are Tumor Suppressors in Choriocarcinoma

These miRNAs include miR-34a and miR-196b, which have been demonstrated to have tumor-suppressor activity in human choriocarcinoma cells [124,125]. The members of the miR-34 family share high sequence homology [169]. Among these, miR-34a is one of the best-known miRNA tumor suppressors and is directly activated by p53 [170,171]. Pang et al. [124] demonstrated that miR-34a suppresses cell proliferation and invasion in choriocarcinoma cells through regulation of the Notch ligand Delta-like one (*DLL1*). Thus, it is possible that, in the future, miR-34a can be used as a therapeutic target for treating choriocarcinoma.

miR-196b has been shown to function as a tumor suppressor in many different cancer types [172,173,174]. A study conducted by Guo et al. demonstrated that miR-196b suppressed proliferation, migration and invasion of human choriocarcinoma cells by inhibiting its transcriptional target *MAP3K1*. miR-196b and MAP3K1 may be considered potential targets for the clinical treatment of hydatidiform mole and possibly human choriocarcinoma [125].

## 7. Conclusions and Future Directions

In summary, multiple lines of evidence suggest that miRNAs are dysregulated in gynecological cancers. The dysregulation patterns, which need to be further confirmed in larger cohorts, could prove to be useful in much-needed applications for diagnosis and prognosis, as well as for therapy prediction. Based on these available and emerging data, miRNAs could have impact in future therapeutic strategies for carcinomas of cervical, endometrial, vaginal, vulval and ovarian origin.

To date, despite improvements in understanding the mechanisms and efficiency of miRNA in therapeutics, there are still particular obstacles to be overcome in order to achieve maximum efficiency. These challenges include: targeted delivery, specificity, stability, immune activation and toxicity in vivo. Once these issues are solved, miRNA therapy will have a major role to play in personalized medicine for various cancers, including RGCs.

GYNOCARE (a European Network for Gynaecological Rare Cancer research: from Concept to Cure) aims to make connections between research (e.g., international, basic, and clinical trials) on RGCs, and the pharmaceutical sector (e.g., focused on innovative, targeted therapies). To bridge the gap between the unmet needs of women afflicted by RGCs and the recent medical and technological advances, both clinicians and their patients need to have good access to current information on possible participation in clinical trials, as well as relevant education and support. For instance, providing a centralized website and medical/coordination assistance may serve as simple steps to accomplish these goals in the clinical setting (e.g., recruitment to research trials, or reinforced adherence to diagnostic and therapeutic management) [5].

## Figures and Tables

**Table 1:** List of deregulated miRNAs in rare gynecological cancers.

Malignancy	Sample Type				References
	Tissue		Cell Line		
	Upregulated	Downregulated	Upregulated	Downregulated	
Clear cell ovarian cancer	miR-9 miR-34a miR-126 miR-509-3-5p miR-509-3p miR-509-5p miR-510	miR-29b miR-449	miR-9	miR-424	[53–57]
Mucinous ovarian cancer	miR-192 miR-194 miR-215				[58]
Ovarian germ cell tumor	miR-122 miR-126 miR-302a miR-302b miR-302c-3p miR-302d miR-371-5p miR-372-3p miR-373 miR-373-3p	miR-199a-5p miR-202-3p miR-214-5p			[68,69]
Granulosa cell tumors of the ovary	miR-10a	miR-29c-3p miR-126 miR-138-5p miR-184 miR-204-5p miR-328-3p miR-501-3p	miR-17 family miR-10a	miR-126	[70–73]
Sex cord stromal ovary tumor	miR-202c-3p miR-513c-5p				[68]
Uterine sarcomas (Leiomyosarcoma, Endometrial stromal sarcoma, Mixed epithelial–mesenchymal tumors)	miR-7-5p miR-34c-5p miR-138-5p miR-196a-5p miR-202-3p miR-210-3p miR-301a-3p miR-335-5p miR-372-3p miR-373-3p	miR-1 miR-1-3p miR-10a-5p miR-23-3p miR-23b miR-125a-5p let-7 family	miR-129-5p miR-141-3p miR-148a-3p miR-202-3p miR-203a-3p	miR-1 miR-1-3p miR-125b-1-3p miR-140-5p miR-152-3p miR-21-5p miR-27b-3p miR-485-5p miR-495-3p	[86–92]
Uterine carcinosarcoma	miR-184	let-7a let-7b-5p let-7d miR-16 miR-26a miR-30c miR-124-3p miR-200 family miR-214		miR-200c	[98,100,101]
Vulvar carcinoma	miR-133a miR-519b	miR-19-b1-5p miR-100-3p miR-223-5p			[107]
Vulvar squamous cell carcinoma	miR-590-5p miR-182-5p miR-183-5p miR-3147 miR-4712-5p	miR-603 miR-103a-3p miR-107	miR-182-5p miR-183-5p miR-223-5p miR-590-5p miR-4712-5p miR-3147	miR-103a-3p miR-107 miR-603	[108–110]
Melanoma of the female genital tract	miR-17-5p miR-19b-3p miR-20a-5p miR-20b-5p miR-146a-5p	miR-15 miR-99a-5p miR-145-5p miR-200a-3p miR-200b-3p miR-494-p miR-1972			[113]
Gestational trophoblastic disease	miR-21 miR-181b-5p miR-181d-5p miR-371a-5p miR-518a-3p miR-519d-3p miR-520a-3p miR-934	miR-199b miR-370-3p miR-517a miR-517b miR-518b miR-519a	miR-21 miR-371a-5p miR-518a-3p	miR-34a miR-196b miR-199b	[120–126]

**Table 2:** miRNAs in serum/plasma of rare gynecological cancer patients.

Malignancy	Serum/Plasma miRNA		References
	Upregulated	Downregulated	
Clear cell ovarian cancer	miR-130a miR-138 miR-187 miR-202		[138]
Ovarian germ cell tumor	miR-302 miR-371~373 Chromosome 19 microRNA cluster (C19MC) miR-371a-3p		[139,140]
Uterine sarcomas (Leiomyosarcoma, Endometrial stromal sarcoma, Mixed epithelial–mesenchymal tumors)		miR-191-5p miR-1246	[141]
Gestational trophoblastic disease		miR-520b miR-520f miR-520c-3p	[142,143]

**Table 3:** Validated mRNA targets and affected pathways of miRNAs relevant in RGCs.

miRNA	Up- or Down-Regulated	Validated Targets	Pathway/Process Affected	Cell Line	References
miR-9	Upregulated	*CDH1*	EMT	OCCC	[55]
miR-10a	Upregulated	*PTEN*	Akt and Wnt pathways	Cancerous granulosa	[73]
miR-21	Upregulated	*PDCD4*	Akt pathway	Choriocarcinoma	[126]
miR-590-5p	Upregulated	*TGFβRII*	TGFβ pathway	VSCC	[108]
miR-3147	Upregulated	*SMAD4*	TGFβ pathway	VSCC	[110]
miR-4712-5p	Upregulated	*PTEN*	AKT, GSK3β and cyclin D1 signaling pathways	VSCC	[109]
miR-34a	Downregulated	*DLL1*	Notch pathway	Choriocarcinoma	[124]
miR-126	Downregulated	*EGFL7*	PI3K/AKT pathway	Cancerous granulosa	[72]
miR-196b	Downregulated	*MAP3K1*	Cell migration and invasion	Choriocarcinoma	[125]

## Data Availability

This review paper does not report any new data.

## Abbreviations

BEP	bleomycin, etoposide, and cisplatin
CSC	Cancer Stem Cell
EMT	epithelial–mesenchymal transition
ESS	endometrial stromal sarcoma
GTD	gestational trophoblastic disease
GTN	gestational trophoblastic neoplasia
hCG	human chorionic gonadotropin
HGSC	high-grade serous ovarian carcinoma
HPV	human papillomavirus
LMS	leiomyosarcoma
mEOC	mucinous ovarian cancer
miRNA/miR	micro-RNA
OCCC	ovarian clear cell cancer
PTEN	phosphatase and tensin homolog deleted on chromosome ten
PTN	persistent trophoblastic neoplasia
RGC	rare gynecological cancer
RISC	RNA-induced silencing complex
TIP	paclitaxel, ifosfamide, and cisplatin
TKIs	tyrosine kinase inhibitors
TRBP	transactivation response RNA binding protein
TsmiRs	tumor suppressors miRs
UCS	uterine carcinosarcoma
UPS	undifferentiated pleomorphic sarcoma
VSCC	vulvar squamous cell carcinoma
3′-UTR	3′ untranslated region
